# Understanding young adult physical activity, alcohol and tobacco use in community colleges and 4-year post-secondary institutions: A cross-sectional analysis of epidemiological surveillance data

**DOI:** 10.1186/1471-2458-10-208

**Published:** 2010-04-26

**Authors:** Nicole A VanKim, Melissa Nelson Laska, Edward Ehlinger, Katherine Lust, Mary Story

**Affiliations:** 1 Division of Epidemiology and Community Health, University of Minnesota, Minneapolis, Minnesota, USA; 2 Boynton Health Service, University of Minnesota, Minneapolis, Minnesota, USA

## Abstract

**Background:**

Young adults experience many adverse health behavior changes as they transition from adolescence into adulthood. A better understanding of the relationships between health promoting and risky health behaviors may aid in the development of health promotion interventions for various types of young adult post-secondary students. Therefore, the purpose of this study was to examine associations between alcohol and tobacco use and physical activity among 2-year and 4-year college students.

**Methods:**

Cross-sectional analyses were conducted using 2007 survey data, collected as part of an on-going post-secondary health surveillance system in Minnesota. Students were randomly selected to participant from 14 Minnesota colleges and universities (six 2-year community and/or technical colleges, eight 4-year post-secondary institutions). The 2007 surveillance data included 9,931 respondents.

**Results:**

The prevalence of demographic characteristics and health behaviors (e.g., physical activity, tobacco use) differed between young adults attending 2-year and 4-year post-secondary institutions; in general, those attending 2-year institutions are representative of more at-risk populations. Overall, higher levels of moderate, vigorous and strengthening physical activity were associated with higher levels of alcohol consumption and lower levels of smoking. In general, despite the disparities in the prevalence of these risk behaviors, the associations between the behaviors did not differ substantially between 2-year and 4-year post-secondary populations.

**Conclusions:**

These findings illustrate links between leading risk behaviors. Interventions targeting multiple risk behaviors among young adults may warrant further consideration. Overall, future research is needed to support and inform young adult health promotion efforts that may be implemented in a wide array of post-secondary institutions.

## Background

Young adulthood, in addition to being the time of transition from adolescence to adulthood, is also an understudied age of increasing negative health behavior choices. In the United States and around the world a large proportion of young people ages 18-24 years attend post-secondary educational institutions [1]. Therefore, colleges and universities provide an important setting for interventions aimed at assisting young adults in developing and maintaining health-promoting behaviors that lower risk of chronic diseases and premature death. Among the leading risk behaviors, insufficient physical activity, tobacco and alcohol use are well-recognized concerns among young adults and leading causes of population-level mortality overall [2,3]. Research has shown a substantial decrease in physical activity (PA) from adolescence into adulthood [4,5], and 18.4% of 18-24 year olds and 21.5% of 25-34 year olds in the U.S. report not engaging in any PA [6]. In addition, nearly quarter of young adults ages 18-24 years in the U.S. currently smoke cigarettes [7] and previous work has shown that 75% of college smokers continue to smoke cigarettes into adulthood [8]. Furthermore, alcohol use is notably high among young adult post-secondary students [9], with college students being at greater risk of binge drinking than those of the same age who do not attend college [10]. Previous research has found that 41.5% of U.S. college students, ages 18-24 years, reported binge drinking at least once in the previous 30 days [11]. Together, these factors underscore the current multifaceted health concerns for young adults in the U.S.

In a recent systematic literature review, a majority of studies showed a negative association between cigarette use and PA among adults [12]. Little work to date has specifically explored these issues among young adult populations. Some research has suggested an inverse association between college athletic involvement and cigarette smoking [13], as well as binge drinking [14], though the evidence is not entirely consistent [15]. Understanding the co-occurrence of and context for these risky health behaviors may make important contributions to the development of effective health promotion interventions specifically targeting young adults.

Overall, young adults experience many adverse health behavior changes as they transition from adolescence into adulthood. Given the impact of increased alcohol and tobacco use and decreased PA on chronic disease risk and premature death, it is important to gain a better understanding of how and if they are related. In particular, there has been limited research on the relationship between risky behaviors and health promoting behaviors among young adults from a wide range of lifestyles, settings and socioeconomic (SES) groups [16]. For example, while much of the research around young adult risk behaviors has focused on students attending traditional 4-year colleges and universities, less work has focused on other populations, such as those attending 2-year community and technical colleges. Across the U.S. and in numerous other international settings, 2-year colleges may offer educational certificate and/or associate's degree programs, as well as vocation and technical training for specific positions in the workforce. Compared to 4-year colleges, 2-year colleges likely have more enrolled students who are from minority groups and low-income backgrounds, factors that put them at greater risk for adverse health outcomes and chronic disease risk [17]. Two-year community and technical colleges may offer a virtually untapped setting for health promotion intervention delivery among at-risk young adult populations; therefore, it is important to gain a better understanding of risk behavior patterning and population needs in 2-year college students, as well as 4-year college students.

To address these gaps in the literature, the purpose of this study was to: (a) examine the association between PA, as a health promoting behavior, and alcohol and tobacco use, as two risky health behaviors, among 2-year and 4-year college students, and (b) examine how these relationships differ between 2-year and 4-year students.

## Methods

In 2007, Boynton Health Service at the University of Minnesota randomly sampled students from the enrollment registries of 14 participating Minnesota colleges and universities, as part of an on-going post-secondary surveillance system in Minnesota (USA). Of these schools, six were 2-year community and/or technical colleges and eight were 4-year post-secondary institutions. Postcards were sent to selected students notifying them of their eligibility to participate in a survey. Reminder postcards and/or emails were sent to all students to encourage participation; participants received at least two invitations to participate. Students were offered small monetary incentives for participation, as well as an opportunity to win one of several lottery prizes. Additional details regarding sampling and survey implementation are publicly available online http://www.bhs.umn.edu/healthdata/results. Due to differences in campus structure and communications, students at 11 schools completed an online survey, while students at three schools complete paper and pencil surveys. Response rates for online surveys was 43.1% (range for different schools: 28.7% to 57.3%) and for paper and pencil surveys was 34.8% (range: 30.4% to 37.3%). Response rates for 2-year schools was 33.6% (range: 28.7% to 44.2%) and for 4-year schools was 45.8% (range: 32.1% to 57.3%). Of the 24,018 students selected to participate, 9,931 completed the survey. The final response rate was 42%.

### Independent variable: Physical Activity

Three types of PA were examined: moderate, strenuous/vigorous and strengthening. Similar to national surveillance systems and other large-scale surveys of this kind, physical activity was assessed by asking: "In the past 7 days, how many hours did you spend doing the following activities? (A) Strenuous exercise (heart beats rapidly); (B) Moderate exercise (not exhausting); (C) Exercises to strengthen or tone your muscles." Examples of strenuous PA included biking fast, aerobics, dancing, running, basketball, swimming laps, rollerblading, tennis, soccer; examples of moderate PA included walking quickly, baseball, easy biking, volleyball, skateboarding, snowboarding; strenuous PA examples included push-ups, sit-ups, weightlifting/training. Six response options included: "None", "Less than 1/2 hour/week", "1/2-2 hours/week", "2 1/2-4 hours/week", "4 1/2-6 hours/week", "6+ hours/week". Calculations of all three types of PA were dichotomized, with low activity being characterized as engaging in ≤ 2 hours/week (reference group) and high activity as ≥ 2 1/2 hours/week. This cutoff was selected to correspond with recommendations from the American College of Sports Medicine and the American Heart Association [18], as well as the 2008 Physical Activity Guidelines for Americans [19] for moderate-intensity PA. Response options provided in the survey did not allow for an exact correspondence with guidelines for vigorous-intensity (75 minutes/week) or muscle-strengthening activities (≥ 2 days/week) [19]; thus the same cutoff was employed for all activity types as the best available comparison.

### Dependent variable: Alcohol Consumption

Drinking frequency was assessed using: "During the past 30 days, on how many days did you use alcohol (beer, wine, liquor)". Seven response choices ranged from "0 days" to "all 30 days". Participants also reported the average number of drinks consumed in a week, with a possible range of 0-99. Binge drinking was assessed using: "Think back over the last two weeks. How many times have you had five or more drinks in one sitting?" Seven response choices ranged from "I do not drink alcohol" or "None" to "10 or more times". For all questions, a drink was defined as a: bottle of beer, glass of wine, wine cooler, shot glass of liquor, or mixed drink.

To assess alcohol consumption, a nominal variable was created with three categories: abstain, light drinking (no binge drinking), binge/heavy drinking. Those who indicated, "I do not drink alcohol" were classified into the abstain category. Indexing was used to calculate average daily alcohol consumption [20]. This method is particularly helpful in this population since college students tend to engage in binge drinking behavior at higher rates, thus it is important to account for binge drinking episodes in the average amount of alcohol consumed per day. Using cutpoints consistent with the Centers for Disease Control and Prevention definitions, light drinking was defined as ≤ 2 drinks/day for males and ≤ 1 drink/day for females; heavy drinking is ≥ 3 drinks/day for males and ≥ 2 drinks/day for females [21]. Those who reported having ≥ 1 binge drinking episode over the last two weeks were classified into binge drinking. The majority of students who reported heavy drinking also reported binge drinking episodes over the last two weeks. As a result, heavy and binge drinking were combined into the heavy/binge category.

### Dependent variable: Tobacco Use

Weekday and weekend cigarette use were assessed using the following question: " [What is the] average number of cigarettes you smoke on a weekday and weekend day? (Note: 1 pack = 20 cigarettes)." Participants could respond with "Not applicable, I do not smoke", or report their average cigarette use between 0-99, for weekdays (Monday-Thursday) or weekend days (Friday-Sunday). Consistent with cutpoints used in previous studies [12], tobacco use was examined using a nominal variable with three categories for both weekend and weekday tobacco use: non-smoker, light smoker (≤ 10 cigarettes), moderate/heavy smoker (≥ 11 cigarettes) (hereafter referred to as "heavy smoker"). Weekday and weekend cigarette use were differentiated since previous studies have found that younger adults are more likely to be non-daily smokers and may have smoking habits that are variable throughout the week [22].

### Covariates

Self-reported covariates included: gender, age, race/ethnicity, current living residence and employment status. Due to small sample sizes, race was categorized as "white" versus any other race category or combination of race categories (non-white) when included in statistical models. In addition, students who reported living in "Residence hall" (n = 2,068) and "Fraternity/sorority" (n = 67) were combined. Students reporting living in "Public/subsidized housing" (n = 71) was combined with "Other" housing (n = 215).

### Statistical analysis

Due to small sample size, participants that reported being transgender (n = 10) were excluded from analysis. Although students were only invited to participate in the survey if they were registered with their college/university as being ≥ 18 years of age, inconsistencies in survey responses were later discovered. To address the issue of outliers, students <15 or >99 years (n = 11) were excluded. Students with missing values were excluded from analyses. Missing data ranged from 0.16% (gender) to 0.50% (age). The final analytic sample size was (n = 9,757). To assess differences between 2-year and 4-year schools across demographic characteristics, unadjusted logistic regression, adjusted for school clustering, was used. Relative risk was estimated using multinomial logistic regression to model the association between the outcomes of drinking and weekday and weekend smoking with PA exposure. This portion of analysis was adjusted for the covariates listed above. School type was included in the model to assess differences between 2-year and 4-year institutions. Interactions between school type and PA were included to assess differences in the relationships between drinking, smoking and PA by school type. Confidence intervals (CI) were adjusted for the clustering nature of the study, using the STATA 'cluster' command option. Statistical analyses were conducted using STATA version 9.0 and 10.1 (STATA Corporation, College Station, TX, 2005). The University of Minnesota IRB approved these analyses.

## Results

### Sample Characteristics

Overall, 37.8% of respondents were male and 62.2% were female. Most participants reported being 19-22 years of age (59.2%), white (86.5%), renting or sharing rent (46.0%) or living in a residence hall or a fraternity/sorority (21.8%), working 0 hours per week for pay (27.5%) or 10-19 hours per week for pay (21.6%) (Table 1). Most also engaged in low levels (i.e., ≤ 2 hours/week) of moderate (59.2%), vigorous (70.3%), and strengthening PA (81.2%), consumed alcohol (80.7%) and did not smoke cigarettes (82.4%) (Table 2). Compared to participants attending traditional 4-year institutions, those attending 2-year community/technical colleges were significantly more likely to be older, live in a parent's home or own a home of their own, work 20-29 hours per week for pay or 40 hours per week for pay, engage in low levels of moderate and vigorous PA, and smoke cigarettes.

**Table 1:** Demographic characteristics of college students by type of school attended, 2007 (n = 9,757)

	2-year school		4-year school		P-value^a^
	N	(%)	N	(%)	
*Gender*					0.08
Male	863	(32.3)	2,825	(39.9)	
Female	1,812	(67.7)	4,257	(60.1)	
*Age*					<0.001
<18 years	190	(7.1)	381	(5.4)	
19-20 years	750	(28.0)	2,394	(33.8)	
21-22 years	437	(16.3)	2,202	(31.1)	
23-24 years	243	(9.1)	745	(10.5)	
25-30 years	453	(16.9)	822	(11.6)	
31+ years	602	(22.5)	538	(7.6)	
*Race/ethnicity*					<0.001
White	2,373	(88.7)	6,066	(85.7)	
African American	64	(2.4)	103	(1.5)	
Asian	65	(2.4)	459	(6.5)	
Hispanic	20	(0.8)	70	(1.0)	
American Indian	49	(1.8)	53	(0.8)	
Other/Mixed	104	(3.9)	331	(4.7)	
*Current residence*					<0.001
Parent's home	804	(30.1)	609	(8.6)	
Rent	988	(36.9)	3,493	(49.3)	
Residence hall	27	(1.0)	2,108	(29.8)	
Own a house	751	(28.1)	691	(9.8)	
Other	105	(3.9)	181	(2.6)	
*Employment Status*					<0.001
0 hours	524	(30.3)	2,148	(19.6)	
1-9 hours	196	(20.3)	1,437	(7.3)	
10-19 hours	497	(22.7)	1,608	(18.6)	
20-29 hours	610	(15.3)	1,083	(22.8)	
30-39 hours	316	(4.8)	339	(11.8)	
40 hours	294	(3.1)	220	(11.0)	
More than 40 hours	236	(3.4)	243	(8.8)	

^a ^Difference between 2-year and 4-year schools; adjusted for school-based clustering

**Table 2:** Physical activity, alcohol and tobacco use among college students by type of school attended, 2007 (n = 9,757)

	2-year school		4-year school		P-value^a^
	N	(%)	N	(%)	
*Physical activity*					
Moderate: ≥ 2.5 hours/week	911	(34.1)	3,078	(43.5)	<0.001
Vigorous: ≥ 2.5 hours/week	627	(23.4)	2,278	(32.2)	<0.001
Strengthening: ≥ 2.5 hours/week	468	(17.5)	1,362	(19.2)	0.40
*Drinking behavior^b^*					0.23
Abstain	607	(22.7)	1,272	(18.0)	
Light (no binge)	1,194	(44.6)	2,976	(42.0)	
Heavy/Binge	874	(32.7)	2,834	(40.0)	
*Weekday cigarette use^c^*					<0.001
Nonsmoker	1,980	(74.0)	6,084	(85.9)	
Light	401	(15.0)	787	(11.1)	
Heavy	294	(11.0)	211	(3.0)	
*Weekend cigarette use^c^*					<0.001
Nonsmoker	1,980	(74.0)	6,084	(85.9)	
Light	342	(12.8)	682	(9.6)	
Heavy	353	(13.2)	316	(4.5)	

^a ^Difference between 2-year and 4-year schools; adjusted for school-based clustering

^b ^Abstain: reported not drinking alcohol; Light (no binge): ≤ 2 drinks/day for males and ≤ 1 drink/day for females; Heavy or binge: ≥ 3 drinks/day for males and ≥ 2 drinks/day for females and ≥ 1 binge drinking episode over the last two weeks

^c ^Nonsmoker: reported not smoking cigarettes; Light: ≤ 10 cigarettes/day; Heavy: ≥ 11 cigarettes/day

### Alcohol use and physical activity

Table 3 highlights the association between alcohol use and PA. After adjusting for school type (i.e., 2-year vs. 4-year), gender, age, race, residence and employment, those who engaged in higher levels of moderate PA were 29% more likely to be heavy/binge drinkers versus abstainers [Adjusted Relative Risk (ARR) (95% CI): 1.29 (1.12-1.49)]. Similarly, vigorous PA [ARR: 1.44 (1.19-1.75)] and strengthening PA [ARR: 1.39 (1.14-1.71)] were associated with increased risk of heavy/binge drinking compared to abstaining. In most models that included an interaction between school type and PA, the interaction was not significant, indicating the association between alcohol use and PA did not vary by school type. For strengthening PA, however, the association with light drinking differed by school type (P = 0.02). Stratified analyses indicate that for participants attending 2-year colleges the association between strengthening PA and light drinking was 1.59 (1.27-2.01), whereas for 4-year students it was 0.96 (0.74-1.25).

**Table 3:** Adjusted^a ^association between drinking behavior, tobacco use and physical activity (n = 9,757), generated via multinomial logistic regression

	Independent variable		
	
Dependent variable	Moderate Physical Activity: ≥ 2.5 hours/week	Vigorous Physical Activity: ≥ 2.5 hours/week	Strengthening Physical Activity: ≥ 2.5 hours/week
*Drinking behavior^b^*			
Abstainers	1.00 (ref)	1.00 (ref)	1.00 (ref)
Light (no binge)	1.13 (0.96, 1.33)	1.15 (0.98, 1.36)	1.11 (0.87, 1.42)
Heavy/Binge	1.29 (1.12, 1.49)	1.44 (1.19, 1.75)	1.39 (1.14, 1.71)
*Weekday smoking^c^*			
None	1.00 (ref)	1.00 (ref)	1.00 (ref)
Light	1.13 (1.00, 1.28)	0.86 (0.75, 0.98)	0.98 (0.80, 1.21)
Heavy	0.95 (0.76, 1.19)	0.58 (0.40, 0.85)	0.68 (0.48, 0.96)
*Weekend smoking^c^*			
None	1.00 (ref)	1.00 (ref)	1.00 (ref)
Light	1.10 (0.95, 1.27)	0.87 (0.74, 1.04)	1.04 (0.85, 1.27)
Heavy	1.06 (0.86, 1.29)	0.63 (0.48, 0.83)	0.67 (0.49, 0.92)

^a ^Adjusted for school type (2-year vs. 4-year), gender, age, race (white vs. non-white), residence type and employment status; Adjusted for school-based clustering

^b ^Abstain: reported not drinking alcohol; Light (no binge): ≤ 2 drinks/day for males and ≤ 1 drink/day for females; Heavy or binge: ≥ 3 drinks/day for males and ≥ 2 drinks/day for females and ≥ 1 binge drinking episode over the last two weeks

^c ^Nonsmoker: reported not smoking cigarettes; Light: ≤ 10 cigarettes/day; Heavy: ≥ 11 cigarettes/day

### Smoking and physical activity

#### Weekday smoking

Results for the association between weekday cigarette use and PA are also presented in Table 3. After covariate adjustment, there was no significant association between weekday smoking and moderate PA. A significant inverse association was present between weekday smoking and vigorous PA, with those engaging in high levels of activity having lower risk of both light smoking [ARR: 0.86 (0.75-0.98)] and heavy smoking [ARR: 0.58 (0.40-0.85)]. There was a similar association between strengthening PA and heavy smoking [ARR: 0.68 (0.48-0.96)], but not light smoking. In assessing the interaction of school type and smoking, there appeared to be differences in the association between both moderate and vigorous PA and heavy weekday smoking across 2-year and 4-year schools (P = 0.08 and P = 0.06, respectively). Stratified analyses indicate that for 2-year college students the association between moderate PA and heavy weekday smoking was 1.15 (0.83-1.61) and between vigorous PA and heavy weekday smoking was 0.79 (0.43-1.48). In contrast, among 4-year students the association between moderate PA and heavy weekday smoking was 0.75 (0.56-1.00) and between vigorous PA and heavy weekday smoking was 0.40 (0.30-0.54).

#### Weekend smoking

Weekend smoking was similarly associated with moderate PA and strengthening PA (Table 3). Increased participation in vigorous PA was not significantly associated with light weekend smoking [ARR: 0.87 (0.74-1.04)], although the direction and magnitude of the association were similar to that of weekday smoking and vigorous PA. The association between heavy weekend smoking and vigorous PA was similar to weekday smoking. The association between weekend smoking and PA did not vary by school type.

## Discussion

In general, the findings of this study suggest that young adults engaging in higher levels of moderate, vigorous and strengthening PA were significantly more likely to be heavy/binge drinkers, regardless of the type of post-secondary institution they were attending. In addition, participants engaging in higher levels of vigorous PA were less likely to engage in any weekday smoking, while higher levels of strengthening PA were associated with decreased risk of heavy weekday smoking only. These relationships existed independent of sociodemographic characteristics, such as gender, age and race/ethnicity.

Overall there were only marginal differences in the association between moderate and vigorous PA and heavy weekday smoking by school type. Among young adults attending 4-year institutions, both moderate and vigorous PA were significantly associated with rates of heavy weekday smoking; however, among those attending 2-year colleges, no significant association was detected. Engaging in higher levels of vigorous PA and strengthening PA was associated with decreased risk of heavy weekend smoking, with no differences by school type.

One unique aspect of this research is its inclusion of a large number of young adults attending 2-year community and technical colleges, who generally represent a more diverse and lower SES group compared to 4-year post-secondary students [23,24]. Despite these sociodemographic differences, the 2-year and 4-year college populations generally yielded similar associations between PA, alcohol and tobacco use. These findings suggest that health behavior messaging and interventions targeting young adults might be adaptable between 2-year and 4-year post-secondary settings. Additional formative research is needed to understand the differences in intervention delivery opportunities across individual campuses, both in the U.S. and around the world.

Previous research has found positive associations between heavier alcohol use and increased physical activity among young adults [25,26], heavier alcohol use has been associated with athlete status and fraternity/sorority membership among 4-year college students [27], and past research has demonstrated the central role of alcohol and tobacco use in social processes among post-secondary organizations [28]. Data on athlete status or fraternity/sorority membership was not collected in this survey. Thus, we were not able to examine such specific contextual differences in the associations between alcohol use and PA. In the U.S. these two social environments of athletics and fraternities/sororities are more prominent at 4-year colleges and universities than 2-year institutions, thus we anticipated this would have further influenced the difference in alcohol use and PA between school type. However, the nonsignificant interaction between PA and school type indicates that the association between high-risk drinking and PA may be explained by factors beyond the school environment. It is likely that the influence of young adult lifestyle norms extends well beyond traditional college campuses and may have a similar impact among highly varied segments of the young adult population.

In addition to the positive associations we observed between alcohol and PA, our findings indicate that there were inverse associations between smoking and PA, which is consistent with previous research [14,29]. Smoking tends to have a negative impact on PA performance, and this may influence active young adults to refrain from smoking. However, motivations behind smoking behaviors may vary among young adults; for example, it is interesting to note that previous research has found that 56% of young adult females felt smoking helped them control their weight [30]. Thus some young adults may be choosing to smoke in an effort to control their weight, rather than engaging in healthy weight control behaviors, such as PA. Additional research is needed to examine the use, adoption and role of weight control behaviors in this population, particularly in relation to other risk behaviors and among lower SES young adult populations where limited research is available.

Finally, while PA may be positively associated with alcohol use and negatively associated with smoking, other research has illustrated a positive association between drinking and smoking among individuals at this age [31]. These findings highlight the complex co-variation that exists among risk behaviors. Given the relationships between these risk factors, there may be reason to believe that interventions aimed at reducing risky behaviors could also include measures to increase health-promoting behaviors. To date, the majority of interventions aimed at young adult college populations tend to focus on single behaviors [32]. Studies examining how increases in PA may impact alcohol and tobacco use have had positive findings, indicating that PA may be an important mediator in maintaining other healthy behaviors [12,14,25,26,33]. Furthermore, a recent multiple behavior intervention utilizing a positive goal of fitness among young adults while addressing multiple health habits such as PA, substance use, dietary habits and sleep patterns found that this type of intervention appears to significantly impact multiple health behaviors [32]. Our results suggest that tobacco prevention/cessation may be another important component for such multi-target intervention strategies. These interventions may also be appropriate for use in 2-year community and technical college settings, where similar patterning of behavior was observed (compared to 4-year colleges), but where absolute rates of risk behaviors appear to be higher.

To our knowledge, this is the first study of its kind to examine associations between young adult alcohol use, tobacco use and PA among such a wide range of post-secondary settings. Despite this strength, several limitations should be considered. The cross-sectional nature of this study limits the ability to draw conclusions on the temporality of these relationships, and longitudinal data are needed. Furthermore, the survey tool used to collect our data may limit our findings. For example, the question on binge drinking episodes does not take into account possible gender differences in the definition of binge drinking [21]. To maximize the efficiency of our needs assessment efforts, much of the survey has been based on single-item indicators of risk. More in-depth assessments of each of the behavioral factors assessed in these analyses likely would reduce error and provide higher validity in characterizing behavioral patterns. In addition, more in-depth assessment methods would allow for more detailed analyses; in our work, we used relatively crude categorizations for behaviors (e.g., physical activity ≥ 2.5 hours/week versus ≤ 2 hours/week), but there may still be a large degree of variability in PA behaviors within each of these categories.

Furthermore, our overall response rate to the survey was 42%, and thus low response rates could have resulted in non-response bias. For example, smokers are known to have lower response rates than nonsmokers. Thus these results may be more conservative than the actual rates in this population. Logistical challenges to working with diverse young adult populations make it difficult for survey-based researchers to achieve higher response rates in the field; nonetheless, this remains a concern that should be noted. Future research should explore mechanisms for improving response rates in the field and/or using statistical methods to correct for possible non-response bias. Finally, participants were drawn from one region of the U.S. and may not represent other geographic regions; additional research is needed to understand how these associations may vary in other international settings.

## Conclusions

In conclusion, there appears to be a positive association between alcohol use and PA and an inverse association between tobacco use and PA among college students. Although the prevalence of health behaviors varied between 2-year and 4-year postsecondary institutions (with young adults attending 2-year community and technical colleges representing a more at-risk population), the magnitude and direction of these associations largely did not differ by school type. Possible explanations may lie in factors that extend well beyond the college campus, such as widespread social norms and other socio-environmental influences among young adults. Interventions targeting multiple health behaviors through a unifying positive health image hold a promise in improving the health of young adult populations. However, additional research on these interventions is needed to determine the most effective way of targeting this population and the leading risk behaviors experienced by this age group, particularly across various international settings.

## Competing interests

The authors declare that they have no competing interests.

## Authors' contributions

NV originated the study, performed data analyses, drafted all sections of text and provided feedback on all drafts of text. ML assisted in data analyses, data interpretation, and provided critical feedback on all drafts of the text. EE oversaw the study design and provided guidance and critical feedback on all drafts of the text. KL assisted in data analyses, led data collection efforts and provided guidance and feedback on all drafts of the text. MS provided guidance and critical feedback on all drafts of the text. All authors read and approved the final text.

## Pre-publication history

The pre-publication history for this paper can be accessed here:

http://www.biomedcentral.com/1471-2458/10/208/prepub
